# Relationship between the microstructural energy release rate of cortical bone and age under compression condition

**DOI:** 10.1038/s41598-024-78819-z

**Published:** 2024-11-08

**Authors:** Ruoxun Fan, Yitong Wang, Zhengbin Jia

**Affiliations:** 1 Automotive Lightweight Engineering Research Center, Yangzhou Polytechnic Institute, Yangzhou, 225127 People’s Republic of China; 2 School of Mechanical and Aerospace Engineering, Jilin University, Changchun, 130022 People’s Republic of China; 3 School of Traffic Engineering, Yangzhou Polytechnic Institute, Yangzhou, 225127 People’s Republic of China

**Keywords:** Microstructural energy release rate, Fracture, Rat femoral cortical bone, Age, Back-calculation, Computational models, Musculoskeletal system

## Abstract

Most studies evaluated the energy release rate of cortical bone macrostructure under Mode I, Mode II, and mixed Mode I-II loading conditions. However, testing the macrostructural energy release rate requires an initial crack and recording the applied load and the corresponding crack length in real-time, which may introduce measurement errors and differences with the actual fracture scenarios. To further understand how the energy release rate contributed to the cortical bone fracture characteristics, this study predicted the microstructural energy release rate of cortical bone and then investigated its age-related varitions. The microstructural energy release rate of femoral cortical bone in rats from different ages was back-calculated by fitting the experimental and simulated load–displacement curves under compression load. The trends in the microstructural energy release rate were revealed, and the underlying reasons for the age-related changes were investigated by integrating the discussion on the cortical bone mechanical parameters at various levels obtained from the previous experiment. The predicted microstructural energy release rate of femoral cortical bone in the rats from 1, 3, 5, 7, 9, 11, and 15 months of age were in the range of 0.08–0.12, 0.12–0.14, 0.15–0.19, 0.25–0.28, 0.23–0.25, 0.19–0.22, and 0.13–0.16 N/mm, respectively. The statistical analyses showed the significant differences in the microstructural energy release rate at different ages. The results indicated an increasing trend followed by a decrease from 1 to 15 months of age, and the correlations between microstructural energy release rate and age were significant. The age-related variations in the microstructural energy release rate may be linked to the changes in the microarchitecture, and the fracture load is influenced by the micro-level mechanical parameters. Notably, the age-related trends in microarchitecture and energy release rate were similar. These findings were valuable for understanding the mechanism underlying the weakening mechanical properties of cortical bone microstructure with age from an energy perspective.

## Introduction

Various factors, such as loading amplitude and constraint conditions, can influence the failure process of cortical bone. However, fracture toughness is the primary mechanical parameter determining its failure characteristics^1^. Fracture toughness is defined by the energy release rate, which controls the crack propagation process and represents the minimum energy required for fracture^2^. The energy release rate serves as a function of geometric, material, and loading parameters, and its value changes with testing and loading conditions^3^.

Most studies evaluate the energy release rate of cortical bone macrostructures under Mode I, Mode II, and mixed Mode I-II loading conditions^4,5^. These tests involve recording the applied load and the corresponding crack length in real-time, but accurately capturing these data, especially during unstable crack propagation, poses significant challenges^6^. Even minor errors in crack length measurement can affect the final result^7^. For example, during complete failure under Mode I, the energy release rate for bovine tibial cortical bone ranges between 0.3 and 1.2 N/mm, while under Mode II, the values range between 0.16 and 0.5 N/mm^8,9^. The energy release rate shows considerable variation even within the same sample, likely due to crack length measurement errors and the differences in loading modes^10^. Furthermore, the testing conditions for the energy release rate of cortical bone macrostructure differed from the actual fracture scenarios^11^. In experiments, an initial crack is often pre-set in the sample, while most real-life bone fractures occur without clear macrocrack beforehand. Therefore, whether the commonly measured energy release rate accurately represents real fracture behavior remains uncertain^12^. To enhance testing accuracy and applicability, a method that avoids pre-setting the initial crack and real-time measurement of the applied load and corresponding crack length should be developed to obtain the energy release rate.

The energy release rate of cortical bone macrostructure is primarily influenced by its geometry and loading conditions, and is also related to the microstructural morphology and material composition^13^. The microstructural energy release rate, which contributes to the macrostructural energy release rate, refers to the average fracture toughness of osteons and corresponds to the element scale in the cortical bone finite element (FE) model^14^. The microstructural energy release rate of cortical bone has two characteristics. First, it remains nearly unchanged with macrocrack propagation, meaning it is independent of loading conditions or geometric factors and primarily determined by the internal material composition and microstructure^15^. As a result, there is no need for a real-time correlation between the applied load and crack length during measurement. Second, because the microstructural energy release rate is a typical mechanical parameter at the micro-level, it does not need to pre-set an initial macrocrack, which allows for simulation of the actual fracture scenarios^16^. These features reduce potential measurement errors from real-time measurement of applied loads and corresponding crack lengths and allow for more accurate modeling of fracture behavior. Consequently, the microstructural energy release rate could accurately reflect the failure characteristics of cortical bone.

Most mechanical parameters, such as bone microstructural morphology and elastic modulus, exhibit age-related changes^17^. The energy release rate of cortical bone macrostructure also varies with age. Some studies indicate that under Mode I, the energy release rate of the cortical bone macrostructure changes slightly with age, while others report a significant decrease under Mode II^18,19^. However, few studies have explored the relationship between the microstructural energy release rate of cortical bone and age. To address this gap, the microstructural energy release rate of cortical bone should be obtained, and changes in the microstructural energy release rate with age can be observed, which would facilitate a comprehensive understanding of the fracture mechanical properties of cortical bone from an energy perspective.

This study built upon our previous experiment on the femoral cortical bone samples from rats of varying ages and conducted fracture simulation for rat femoral cortical bone to back-calculate the microstructural energy release rate. The energy release rate of an element in the cortical bone FE model always remains constant during loading, so the physical significance of the microstructural energy release rate represents the minimum energy required for element failure. The changing trends in the microstructural energy release rate with age were then explored, and the factors contributing to these changes were investigated by incorporating the cortical bone mechanical parameters at various levels obtained from our previous experiment on rats of different ages. Finally, the mechanism underlying the deterioration of the mechanical properties in cortical bone microstructure with age was investigated from an energy perspective.

## Materials and methods

### Previous experiment statement

**Fig. 1:**
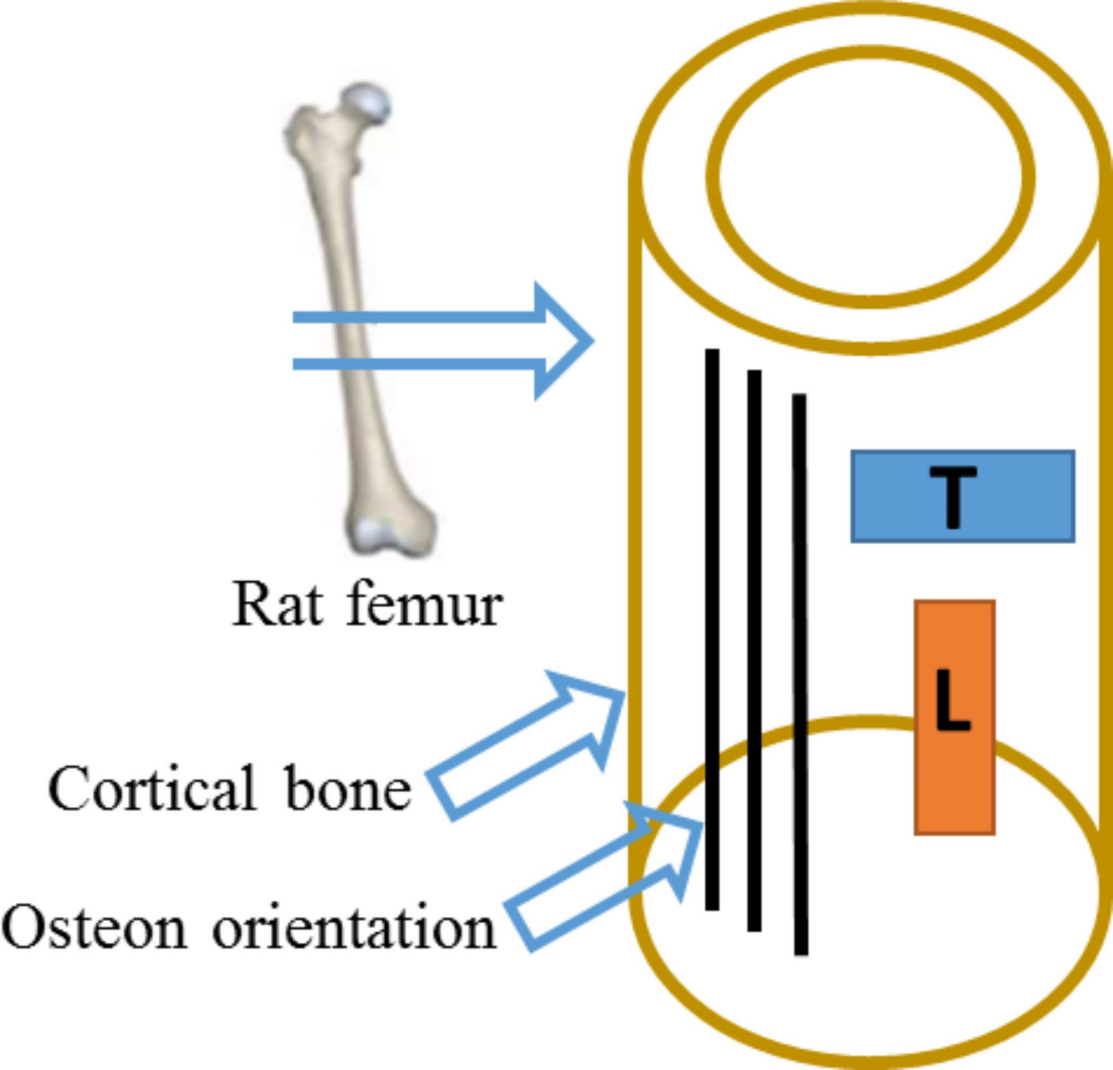
Schematic view of the cortical bone structure with respect to osteon orientation (L-Longitudinal, and T-Transverse directions).

Previous experimental data were utilized to conduct the fracture simulation to back-calculate the microstructural energy release rate of rat femoral cortical bone in this study. The multiscale mechanical parameters of cortical bone from rats of varying ages were measured in the previous experiment^20^. The cortical bone samples were taken from the middle section of the femur. The location of mid-diaphysis was set as a benchmark, and a longitudinal cortical bone specimen with a height of 5 mm was cut along the femoral shaft axis from the femur shaft. The schematic view of the cortical bone structure could be seen in Fig. 1. Given the similarity between the loading direction of cortical bone under compression and the physiological loading direction of the femur, compressive load–displacement curves were obtained at the macro-level in the previous experiment. At the micro-level, the focus was on the changes in cortical bone microstructural morphology and fracture surface with age. Additionally, the cortical bone elastic modulus and mineral grain size were measured at the nano-level. For specific experimental details, please refer to the literature^20^.

### Development of the cortical bone finite element model

In this study, the femoral cortical bone FE models from rats aged 1, 3, 5, 7, 9, 11, and 15 months were established. For each age group, four FE models were developed, leading to a total of 28 models used in the fracture simulations. Each FE model was developed on the basis of its actual micro-CT images that were still derived from the previous experiment. The micro-CT images of femoral cortical bone were imported into the MIMICS to establish the geometric model. Then, the 5 mm-long geometric model was imported into ABAQUS, where C3D4 elements were used to create the three-dimensional (3D) FE models of rat femoral cortical bone. FE models within the same age group exhibited minimal geometric variation due to the consistent sampling location and a uniform height of 5 mm. However, the FE models in varying age groups showed differences in cortical cross-sectional area and thickness, both of which increased progressively with age. Additionally, cortical porosity changed with age. In the period of skeletal maturity (1–7 months of age), cortical porosity decreased linearly while increasing linearly in the second phase (9–15 months of age). For more detailed data on the previous experiment, please refer to the literature^20^.

**Fig. 2:**
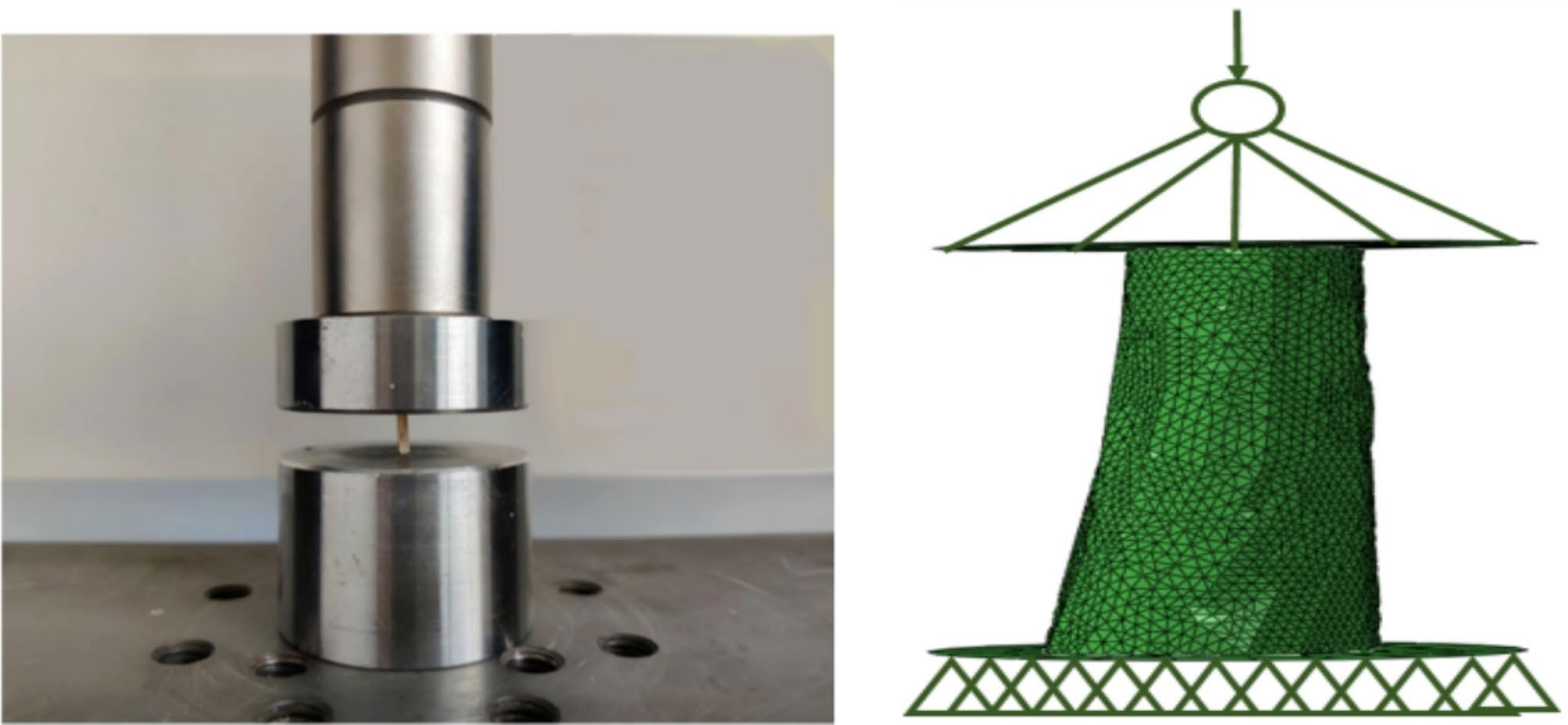
Schematic diagrams of the boundary conditions in the previous experiment and the fracture simulation in this study.

Rigid circular plates were placed above and below the cortical bone to replicate the compression condition in the previous experiment. In the previous experiment, given the larger area of rigid plates compared to the cortical bone surface and the three-time preload, no lateral slip occurred between the plates and the sample during compression. Based on these, the lower surface of the cortical bone was set to “TIE” to the lower rigid circular plate, and the upper surface was set to a frictionless contact with the upper rigid circular plate^3,4^. Additionally, a reference point was established above the press head to apply the load. Simultaneously, all degrees of freedom of the lower rigid circular plate were constrained to complete the boundary conditions. The loading direction was parallel to the oseton orientation. Figure 2 visually compares the cortical bone fracture model established in this study with that from the previous experiment.

### Fracture simulation process

This study aimed to back-calculate the microstructural energy release rate of rat femoral cortical bone by fitting the experimental and simulated load–displacement curves. The failure process was modeled using the continuum damage mechanics theory. While this numerical method had been established, recent studies by our team have demonstrated its suitability for simulating cortical bone fracture^21,22^. On the premise of an appropriate damage variable expression, the continuum damage mechanics model can accurately simulate cortical bone fracture and achieve desirable convergence. Given the predominantly brittle fracture behavior of rat femoral cortical bone under compression, the failed elements in the FE model mainly depend on the element stiffness, failure strain, and microstructural energy release rate. Accordingly, the damage variable expression was set as follows^23,24^:

1 $$\:\text{D}=0\:({{\upepsilon\:}}_{\text{T}}<{{\upepsilon\:}}_{\text{f}\text{t}});\:\text{D}=1-\left(\frac{{{\upepsilon\:}}_{\text{f}\text{t}}}{{1+{\upepsilon\:}}_{\text{T}}}\right){\text{*}\text{e}}^{\frac{\left(1-\frac{{{\upepsilon\:}}_{\text{T}}}{{{\upepsilon\:}}_{\text{f}\text{t}}}\right)\left(\text{C}\text{*}{{{\upepsilon\:}}_{\text{f}\text{t}}}^{2}\text{*}{\text{L}}_{\text{c}}\right)}{\text{G}}}\:({{\upepsilon\:}}_{\text{T}}\ge\:{{\upepsilon\:}}_{\text{f}\text{t}})$$

2 $$\:\text{D}=0\:({{\upepsilon\:}}_{\text{C}}<{{\upepsilon\:}}_{\text{f}\text{c}});\:\text{D}=1-\left(\frac{{{\upepsilon\:}}_{\text{f}\text{c}}}{{1+{\upepsilon\:}}_{\text{C}}}\right){\text{*}\text{e}}^{\frac{\left(1-\frac{{{\upepsilon\:}}_{\text{C}}}{{{\upepsilon\:}}_{\text{f}\text{c}}}\right)\left(\text{C}\text{*}{{{\upepsilon\:}}_{\text{f}\text{c}}}^{2}\text{*}{\text{L}}_{\text{c}}\right)}{\text{G}}}\:({{\upepsilon\:}}_{\text{C}}\ge\:{{\upepsilon\:}}_{\text{f}\text{c}})$$

Where D is damage variable; $\:{{\upepsilon\:}}_{\text{C}}$ is compressive strain; $\:{{\upepsilon\:}}_{\text{T}}$ is tensile strain; $\:{{\upepsilon\:}}_{\text{f}\text{c}}$ is critical compressive failure strain; $\:{{\upepsilon\:}}_{\text{f}\text{t}}$ is critical tensile failure strain; $\:\text{C}$ is element stiffness in the loading direction; $\:{\text{L}}_{\text{c}}$ represents element characteristic length; G is microstructural energy release rate.

**Fig. 3:**
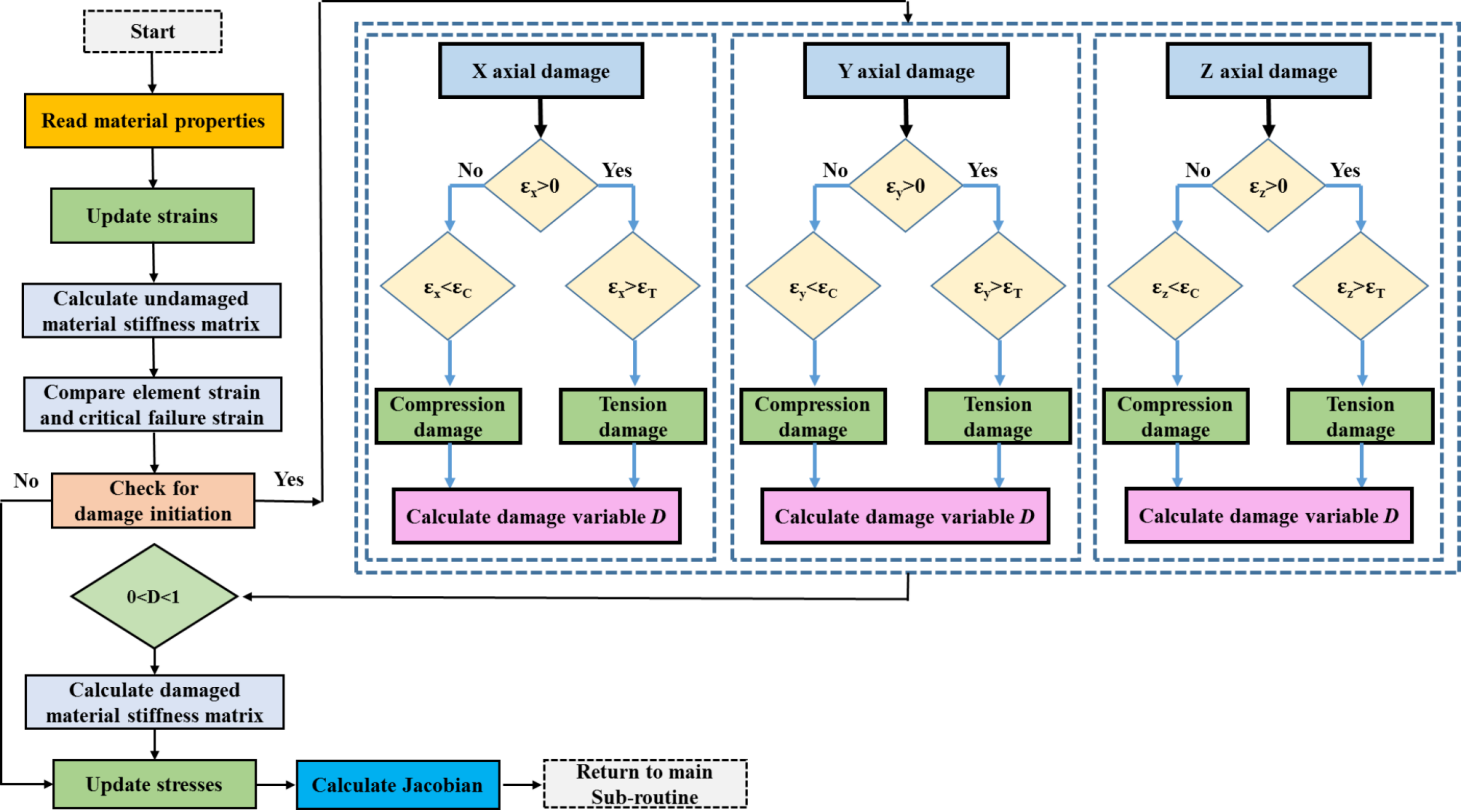
The fracture simulation flow in the cortical bone finite element model.

This study developed a UMAT subroutine in ABAQUS to simulate fracture behavior. When the element strain was less than the corresponding critical failure strain, the element was considered undamaged, and the initial value of damage variable *D* was set to 0. As the compressive load increased, the element was deemed damaged once its strain exceeded the critical failure strain. According to Eq. (1) or (2), the stiffness of the damaged element decreased until the damage variable *D* reached 0.999, indicating complete failure. When a large number of elements could no longer bear the load, the apparent fracture occurred, resulting in the apparent load–displacement curve. Importantly, this program allowed for tensile or compressive damage in the elements based on loading directions and damage occurrence in the X, Y, and Z axes by comparison of the element strain with the critical failure strain. As a result, the damage direction was automatically determined. Figure 3 illustrates the detailed simulation process.

### Fitting process for the microstructural energy release rate

The material input parameters of the FE models, including the longitudinal and transverse elastic moduli of the rat femoral cortical bone, have been obtained in the previous experiment^20^. The critical tensile and compressive failure strains of the rat femoral cortical bone were also determined in the earlier study^25^. The initial element stiffness in the loading direction can be derived from the elastic modulus (Table 1). The characteristic length (L_c_) of elements, typically calculated as the cube root of the element volume for 3D elements, could be automatically computed during iteration^26^. Consequently, the microstructural energy release rate of the element in the FE model was the only unknown material parameter in the Eqs. (1) or (2). The predicted load–displacement curve could be fitted with the experimental curve by adjusting the value of the microstructural energy release rate during simulation. The fitting process began with an initial microstructural energy release rate of 0.01 N/mm, increasing by 0.01 N/mm in each simulation until the predicted curve successfully matched the experimental curve, as indicated by a difference of less than 5% in the fracture load. The assigned value at this point represented the microstructural energy release rate for the elements.

**Table 1:** Material parameters in the simulation measured from the earlier studies^20,25^.

	1-month	3-months	5-months	7-months	9-months	11-months	15-months
The average longitudinal elastic modulus of rat femoral cortical bone (MPa)	5821	10,365	21,964	30,143	31,057	31,243	30,660
The average transverse elastic modulus of rat femoral cortical bone (MPa)	4727	8085	17,571	23,067	24,456	24,197	24,510
The average critical tensile failure strain of rat femoral cortical bone material	2.35%	2.61%	2.67%	2.79%	2.47%	2.15%	1.89%
The average critical compressive failure strain of rat femoral cortical bone material	3.92%	4.35%	4.48%	4.65%	4.11%	3.58%	3.15%
Poisson’s ratio	0.3	0.3	0.3	0.3	0.3	0.3	0.3

### Statistical analysis

All statistical analyses were performed using SPSS 19.0 software. The age-related differences in the microstructural energy release rate were analyzed using one-way analysis of variance (ANOVA). Linear regression analyses were performed to test the correlations between the age and microstructural energy release rate. Correlations were reported as the square of the Pearson’s correlation coefficient (r^2^), and the statistical significance level was 0.05.

## Results

### Mesh sensitivity analysis

**Fig. 4:**
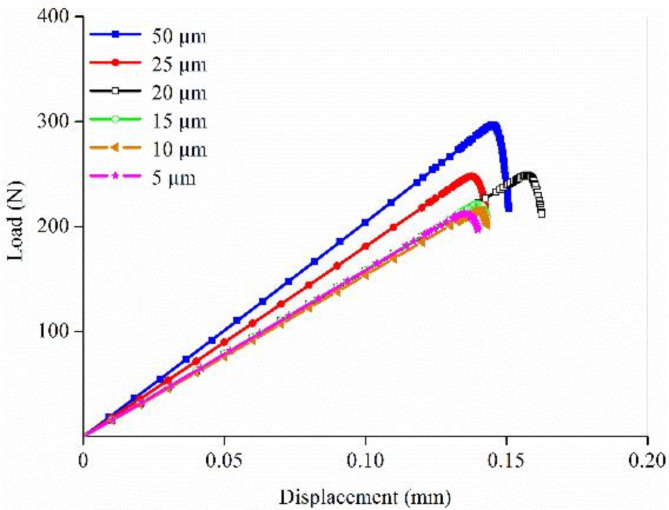
Mesh sensitivity analysis of the finite element model of 1-month-old cortical bone.

Element size significantly influences crack propagation during fracture simulation, necessitating a sensitivity analysis to identify the appropriate range of element sizes in the FE models. Using the FE model of 1-month-old cortical bone as an example, the element size was initiated at 50 μm and decreased in increments of 5 μm until the load–displacement curve converged. The convergence criterion was defined as less than 5% variation in the predicted fracture loads among the models with different element sizes^27^. As shown in Fig. 4, the curves predicted by various mesh models exhibited similar shapes, indicating that while element size affected the predicted results, it had minimal impact on the failure mechanism. The fracture load did not show a linear relationship with mesh refinement. During the mesh refinement within 20 μm, the curves converged, especially in the mesh range of 10–15 μm, where the differences in fracture loads were less than 5%. Given that the fracture simulation method employed in this study prevented crack propagation through elements, a small element size was required. Therefore, the element size for all 28 FE models of rats at different ages was standardized at 10 μm.

### Fracture simulation accuracy analysis

**Fig. 5:**
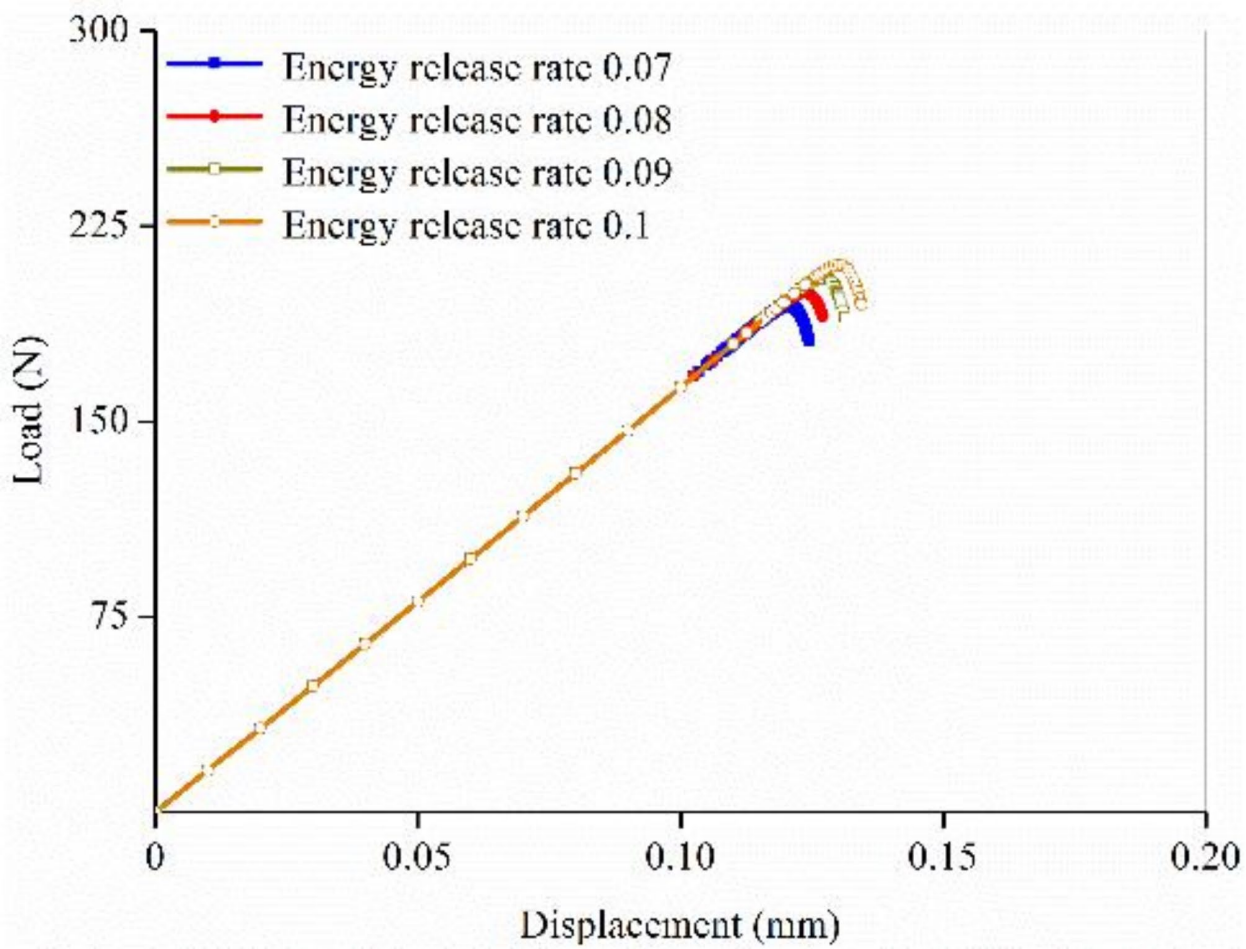
Prediction accuracy analysis on the fracture simulation.

Since all other material input parameters in the FE models were fixed, the failure process was primarily controlled by the microstructural energy release rate of the elements. To evaluate the accuracy and monotonic uniqueness in the fitting process, a sensitivity analysis was performed. Using the FE model of 1-month-old cortical bone as an example, the effects of the microstructural energy release rate on the fracture loads were analyzed. Figure 5 illustrates that for every increment of 0.01 N/mm in the microstructural energy release rate, the fracture load increased by approximately 5 N. This finding indicated that adjusting the microstructural energy release rate in increments of 0.01 N/mm can control the prediction accuracy of the cortical bone fracture load within approximately 5 N. Additionally, as the energy release rate increased, the predicted fracture load exhibited a monotonic increase without any decrease, demonstrating good convergence throughout the fitting process.

### Comparisons between the simulated and experimental data

**Fig. 6:**
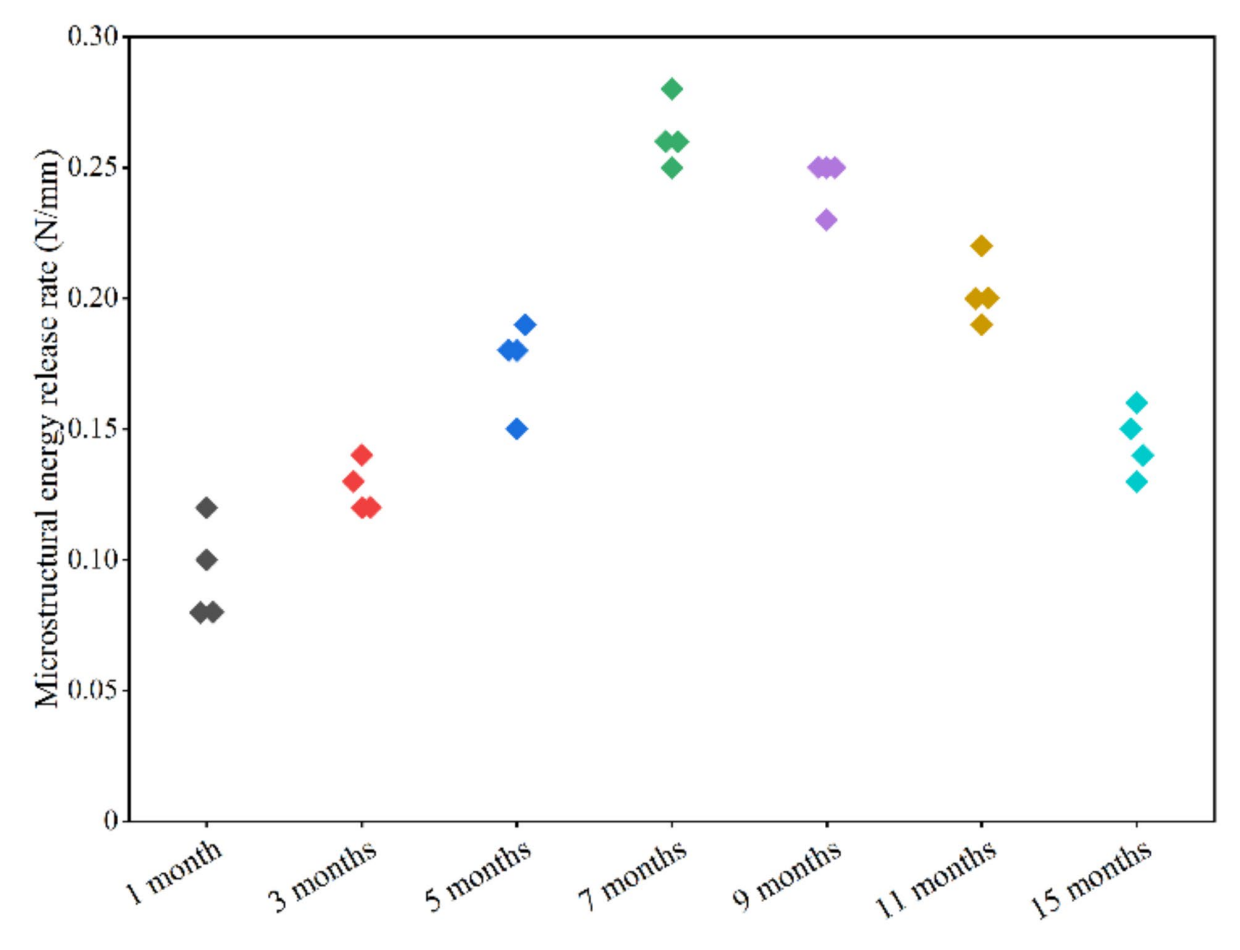
Microstructural energy release rate of the cortical bone in rats with different months.

Figure 6 exhibits the predicted microstructural energy release rate of cortical bone in rat femurs at different ages. Each age group consisted of four cortical bone FE models. The variations in the microstructural energy release rate with age were biphasic. In the first phase (1–7 months of age), the microstructural energy release rate increased linearly, reaching a peak at 7-months-old, and the correlations between microstructural energy release rate and age were significant in this phase (y = 33.133x − 1.467, r^2^ = 0.911, *p* < 0.05). In the second phase (9–15 months of age), the microstructural energy release rate decreased linearly, and the correlations with age were significant (y = − 56.943x + 22.913, r^2^ = 0.930, *p* < 0.05). The statistical analyses showed the significant differences in the microstructural energy release rate at different ages. The microstructural energy release rate in 1 month of age was significantly lower than that in other ages (*p* < 0.05). No statistical difference was found in the microstructural energy release rate between 3 and 15 months of age, but they were significantly lower than others except 1 month of age (*p* < 0.05). The microstructural energy release rate in 5 months of age was significantly greater than that in 1, 3, 15 months of age, and significantly lower than that in 7, 9, 11 months of age (*p* < 0.05). No statistical difference was found in the microstructural energy release rate between 7 and 9 months of age, and they were significantly greater than that in other ages (*p* < 0.05).

**Fig. 7:**
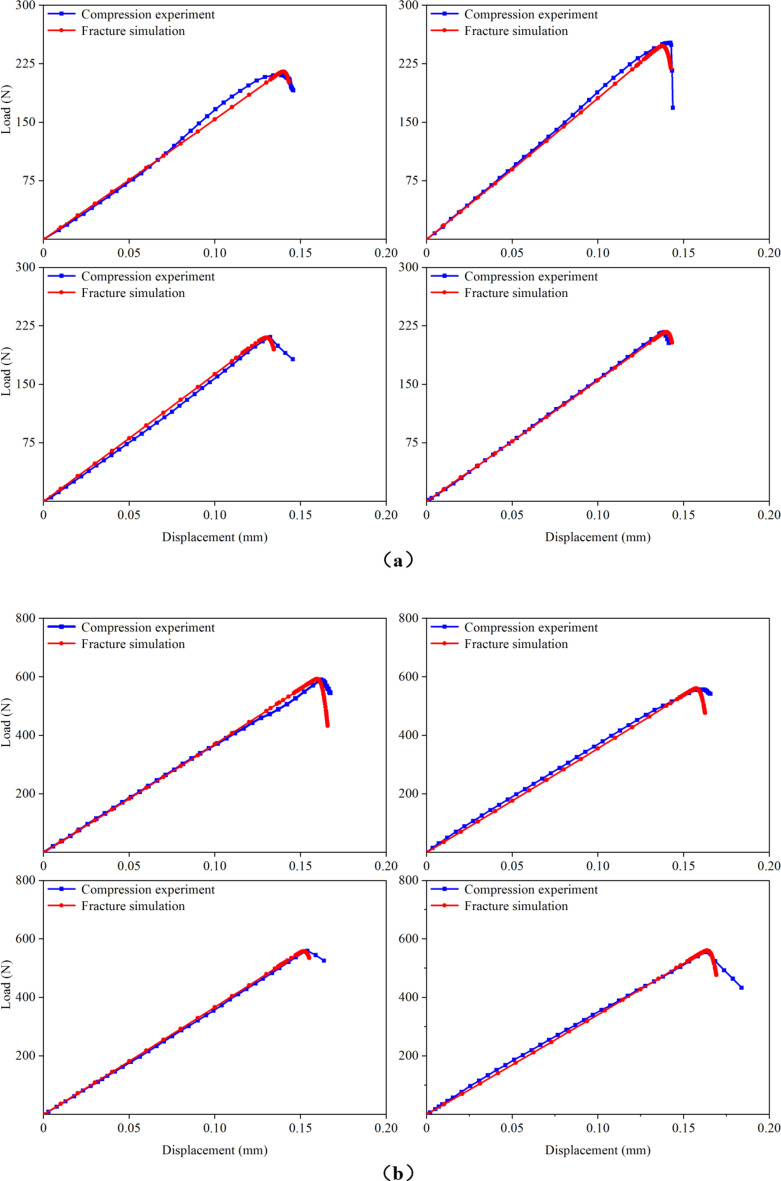

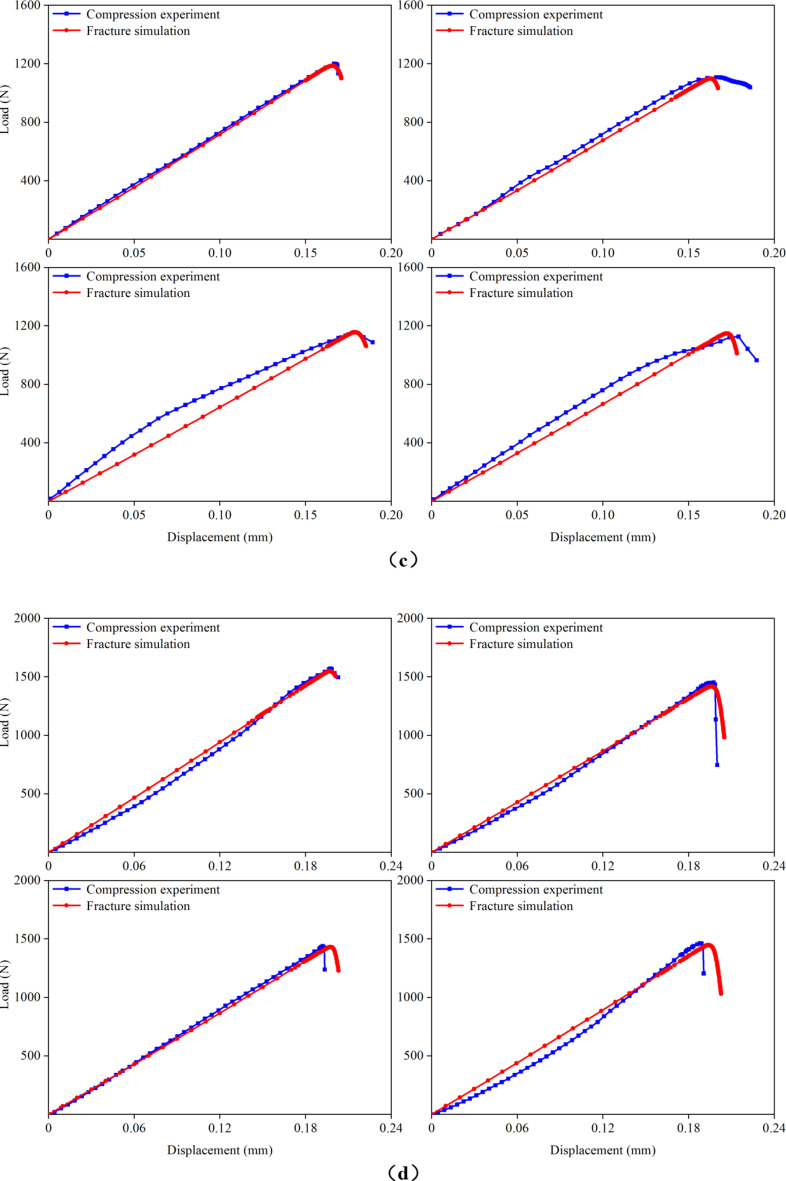

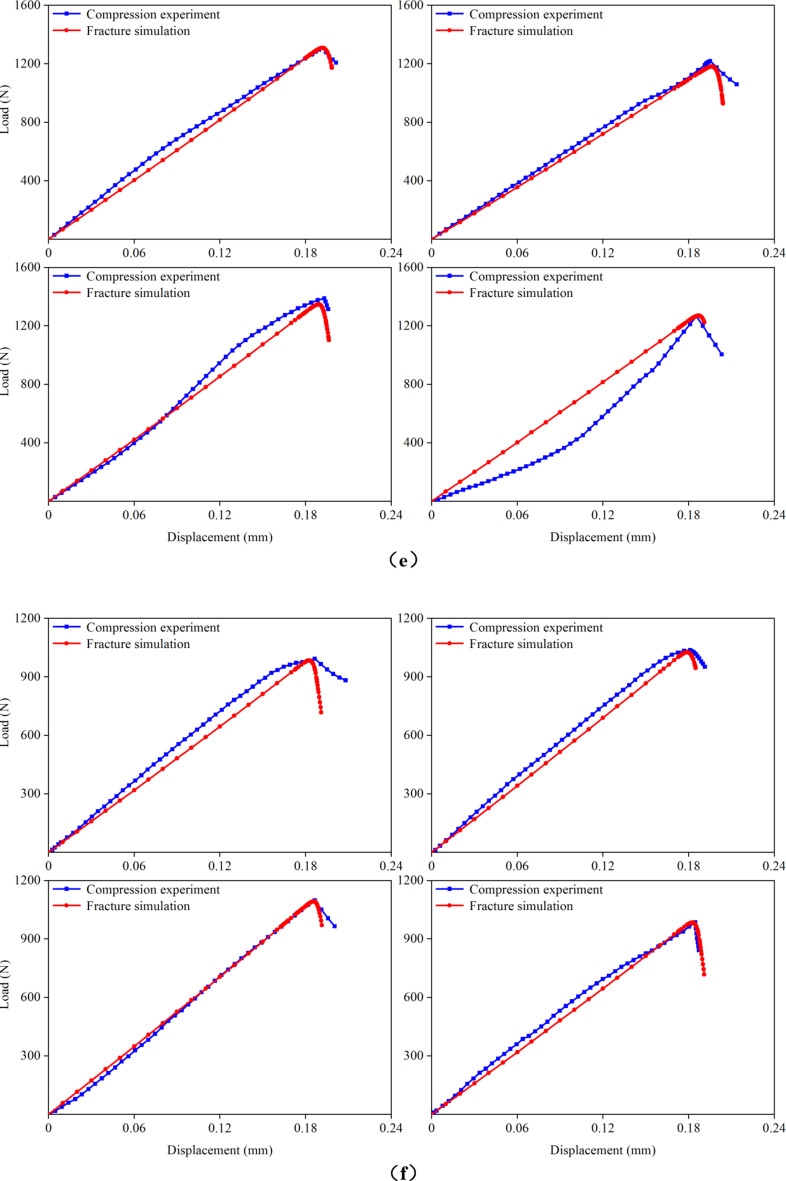

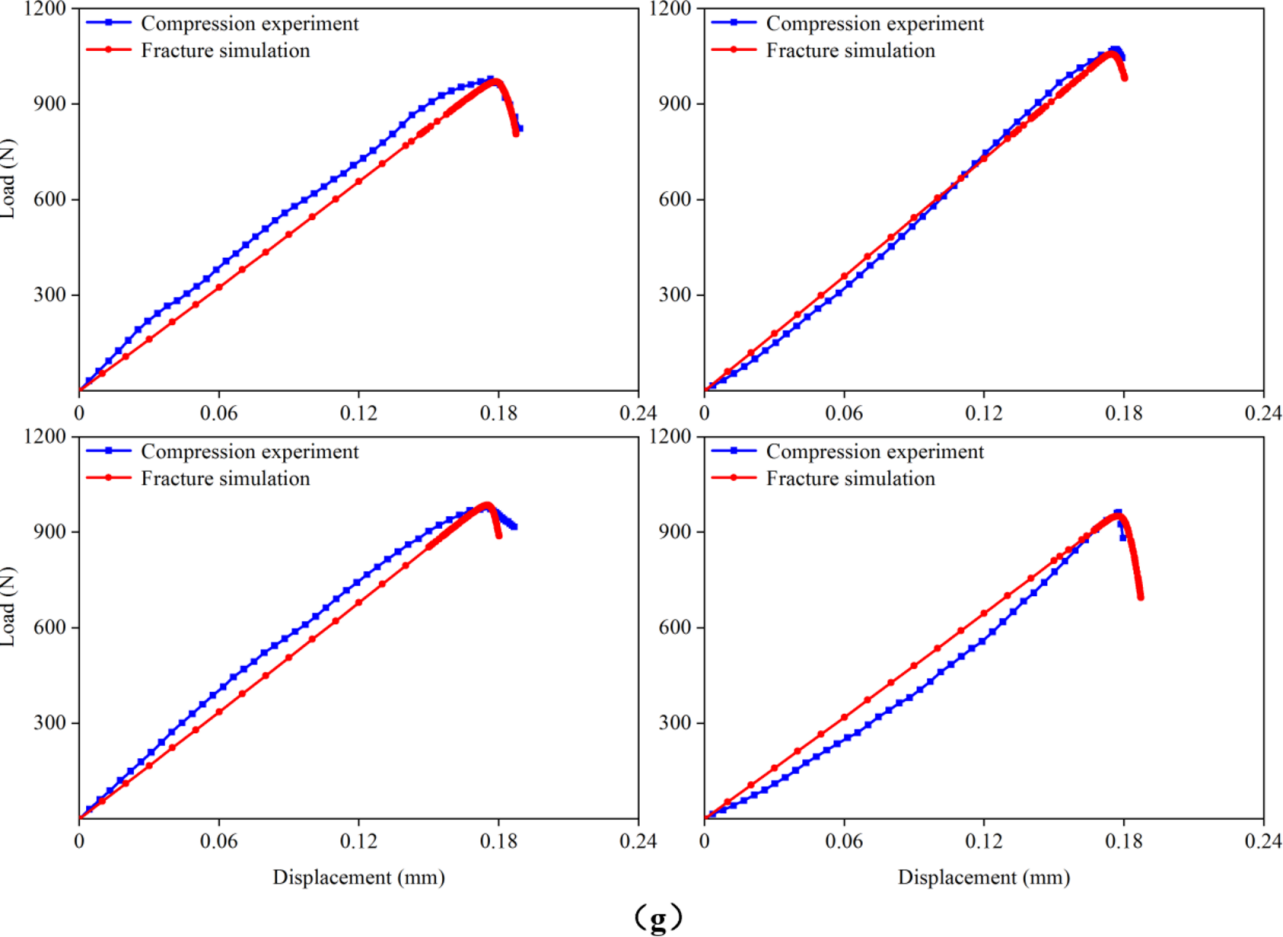
Comparison of the load–displacement curves in the previous experiment and this fracture simulation. (**a**) 1-month-old; (**b**) 3-months-old; (**c**) 5-months-old; (**d**) 7-months-old; (**e**) 9-months-old; (**f**) 11-months-old; (**g**) 15-months-old.

Figure 7 presents the load–displacement curves for the cortical bones of rat femurs at 1, 3, 5, 7, 9, 11, and 15 months of age, obtained from both compression experiments and fracture simulations. The experimental curves were derived from the previous experiment^20^. For each group, four FE models were simulated, resulting in a total of 28 samples that underwent fracture simulations. When the microstructural energy release rate was appropriately selected, the predicted load–displacement curves closely matched the experimental curves. In this study, successful fitting was defined as achieving a less than 5% difference between the predicted and experimental fracture loads. Analysis of the load–displacement curves revealed that the femoral cortical bone did not exhibit a distinct yield stage during compression, directly entering the fracture stage instead. This observation was crucial for accurately applying the fracture simulation method employed in this study. Additionally, the comparison of the load–displacement curves highlighted discrepancies between the apparent elastic moduli obtained from the simulation and experiment. These differences arose because the elastic modulus for the same age group of cortical bones was assigned an average value from the previous experiment, leading to variations between the assigned elastic moduli and actual values for the FE models.

**Fig. 8:**
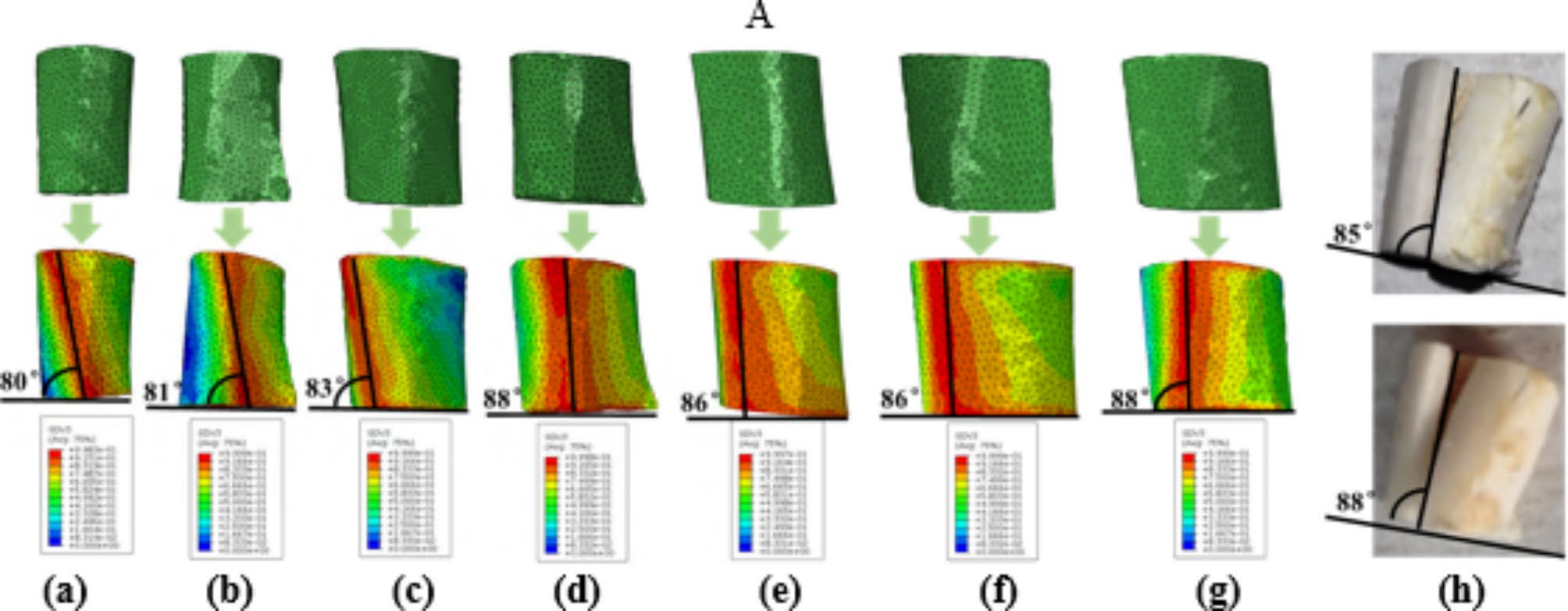
Comparison of the fracture patterns in the rat femoral cortical bone. (**a**) 1-month-old; (**b**) 3-months-old; (**c**) 5-months-old; (**d**) 7-months-old; (**e**) 9-months-old; (**f**) 11-months-old; (**g**) 15-months-old; (**h**) Experimental fracture sample.

Figure 8 exhibits the fracture pattern in the cortical bone of rat femurs at different ages, comparing the fracture simulations with previous experimental findings. Initially, microcracks predominantly appeared on the upper and lower surfaces of the FE models. These microcracks subsequently coalesced to form a complete crack that traversed the entire structure, leading to an apparent fracture. The experimental fracture patterns corroborated this, displaying a distinct macrocrack running through the structure, along with additional microcracks and localized compressive deformations on the upper and lower surfaces. The macrocrack in the experimental structure formed an angle of 85° to 90° with the horizontal direction. The simulation showed that the cortical bone structure at different ages primarily experienced longitudinal (Z-axial) cracking under compression, with the crack propagating mainly aligned with the loading direction. The angle between crack propagation and the horizontal direction varied with age. As the geometric morphology of the cortical bone structure changed with age, leading to differences in high-strain areas that influenced the angles of crack propagation. At younger ages, the angle was relatively small, but as cortical thickness increased with age, the angle gradually approached vertical alignment, as depicted in Fig. 8. Overall, both the fracture path length and the crack propagation angles were consistent with the fracture patterns observed in the previous experiment.

## Discussion

Most studies have measured the energy release rate of cortical bone macrostructure, which requires an initial crack and real-time measurement of the applied load and corresponding crack length^28,29^. To enhance testing accuracy and convenience, this study developed an approach that utilized experimental data and fracture simulation to predict the microstructural energy release rate of cortical bone in rats at different ages. This method eliminated the need for an initial crack and can predict the microstructural energy release rate across various types of cortical bone structures. The microstructural energy release rate remains nearly constant in osteon, regardless of the loading process. This contrasted with the macrostructural energy release rate, which changes with crack length, thereby facilitating discussion on the changes in the fracture mechanical properties with age.

This study focused on investigating the age-related changes in the microstructural energy release rate of rat femoral cortical bone structure. The predicted results indicated a gradual increase in the microstructural energy release rate from 1 to 7 months of age, followed by a decline from 9 to 15 months of age, and the statistical analyses showed the significant differences in the microstructural energy release rate at different ages. This trend differed from the changes in the tissue elastic modulus with age, but aligned with the reported trend of the macrostructural energy release rate with age under Mode II^30,31^. Since the microstructural energy release rate and tissue elastic modulus are typical micro-level mechanical parameters, the elastic modulus had minimal influences on the microstructural energy release rate. Consequently, the variations in the microstructural energy release rate with age may be related to the changes in the microarchitecture, including cortical bone density, porosity, and mineral content.

The microarchitecture features of the fractured surface detected by SEM in the previous experiment revealed that from 1 to 7 months of age, the crystals exhibited a plate-like texture with clear continuity of the mineral phase in bone samples. As the crystals grew transversely along the collagen fibers from 9 to 15 months, they became elongated, and parts of the plate texture were pelt off, resulting in a mixed fibrous-plate texture^20^. Additionally, the calcium and phosphorus contents in cortical bone significantly increase from 1 to 7 months of age and remain unchanged from 9 to 15 months of age, as reported^20^. Therefore, the changes in cortical microarchitecture and mineral content may explain the rapid increase in the microstructural energy release rate from a young age to maturity. With aging, the crystals became disordered, and the collagen fibers were thinner and more fragile, decreasing the osteon numerical density and weakening the osteon morphology^20,32–34^. As reported in the literature, the crack initiation typically involves multiple microcracks connected by the main crack. The main crack often arrests or deflects upon encountering an osteon. Crack propagation through the several layers in an osteon is challenging and often results in the formation of a series of microcracks. These microcracks eventually link, causing the crack to deflect and propagate around individual osteons, producing a semi-circular crack path^35,36^. Thus, osteon density and morphology may play a crucial role in crack arrest. As osteon density decreased and its morphology weakened with age, the ability of cortical bone to arrest cracks diminished, reducing the resistance to fracture. In this study, these age-related changes resulted in a more pronounced brittle fracture pattern and a progressive decrease in the fracture load.

These conditions led to a significant decrease in the microstructural energy release rate from 7 to 15 months of age. However, the morphology of the mixed fibrous-plate mineral crystal in older rats remains superior to the mineral crystal in younger rats, so the previous experiment indicated that the cortical porosity at 15-months-old was lower than that at 1-months-old^20,37^. In addition, no significant decrease in the calcium and phosphorus contents in the cortical bone occurs from 7 to 15 months of age, and the cortical bone mineral grains in older rats were relatively larger than those in younger rats^20^. Therefore, the microstructural energy release rates of cortical bone at 11 and 15 months of age remained greater than those at 3-months-old. This phenomenon suggested that the fracture mechanical properties of cortical bone in older rats were superior compared to those in younger rats.

The fracture load gradually decreased from 7 to 15 months of age, while the cortical porosity increased and the tissue elastic modulus remained relatively stable. The strength limit, which is a critical factor in determining fracture, is primarily influenced by both the elastic modulus and bone quality^38,39^. Since the elastic modulus showed minimal decline during this period, the reduced osteon density and microstructural energy release rate may contribute to the observed decrease in fracture load with age. Previous studies have reported a positive correlation between fracture toughness and ultimate strength, and the results in this study aligned with those findings^40,41^. However, the relationships among the fracture toughness, elastic modulus, and osteon density have not been extensively investigated. This study highlighted the substantial impact of microstructural energy release rate on the cortical bone strength limit, and further implied a slight correlation between the elastic modulus and microstructural energy release rate because they cannot mutually influence each other. Furthermore, by combining the predicted results from this study with previous experimental data, it is evident that the trends in the microstructural energy release rate were similar with the varitions in osteon density and cortical porosity with age. This suggests a strong correlation between the microstructural energy release rate and bone density. In summary, the fracture load in cortical bone structure would be influenced by the micro-level mechanical parameters, such as elastic modulus, microarchitecture, and energy release rate. Notably, the changing trends in bone density and energy release rate were similar, while both differed from the change in the elastic modulus. Therefore, when the geometry morphology of cortical bone structure was fixed, clinical fracture prevention should not focus solely on the elastic modulus^42^. The changes in the microstructural energy release rate and cortical density should also be considered, and the changing trends in these two parameters tend to be consistent. These findings were valuable for investigating the mechanism underlying the weakening mechanical properties of cortical bone microstructure with age from an energy perspective, and informing more comprehensive strategies for preventing fractures in clinical practice.

Although the microstructural energy release rate of cortical bone in rat femurs was predicted, several limitations were encountered. First, due to the limited number of test samples in the previous experiment, only four cortical bone FE models were developed for each age group, resulting in difficulty in obtaining an average value for the microstructural energy release rate. To address this limitation, the microstructural energy release rate for each model was individually listed, and the discrepancy in value for each model in the same month was not great. Second, this study primarily focused on the microstructural energy release rate under compression, whereas the actual fracture loading environment is more complex and involves various types of loads, such as torsion and bending^43^. Thus, restricting the analysis to compression alone is insufficient for fully understanding the relationship between energy release rate and fracture behavior. Hence, further investigation is planned to examine the influence of different loading conditions on the microstructural energy release rate of cortical bone in rat femurs at different ages.

## Conclusions

This study predicted the microstructural energy release rate of rat femoral cortical bone at different ages. The variations in the microstructural energy release rate with age were related to the changes in the microarchitecture. The fracture load in cortical bone structure is influenced by the micro-level mechanical parameters, including elastic modulus, microarchitecture, and energy release rate. Notably, the trends in bone density and energy release rate were similar, while both differed from the changes in the elastic modulus. These findings were crucial for understanding the mechanism underlying the weakening mechanical properties of cortical bone microstructure with age from an energy perspective, and informing more comprehensive strategies for preventing fractures in clinical practice.

## Data Availability

The datasets used and/or analysed during the current study available from the corresponding author on reasonable request.
